# Essay on the Origin of the Epidemical Fever in Spain, with an Abridged Plan for Its Cure, and Some Proposals to Prevent Both Progress and Return of Similar Epidemics

**Published:** 1805-02-01

**Authors:** W. Domeier

**Affiliations:** Physician to the Forces


ESSAY ON THE ORIGIN OF THE EPIDEMICAL FEVER IN SPAIN, *with an abridged Plan for its Cure, and some Proposals to prevent both Progress and Return of similar Epidemics. By* W. DOMEIER, M.D. *Physician to the Forces.*

THE investigation of the cause of the epidemic, now ravaging the south of Spain, Marseilles, and Leghorn, is certainly a matter of the utmost importance, as well to the speculative philosopher and practical physician, as to the inhabitants who labour under this horrid scourge, because it is the only way to find out an infallible cure. I venture therefore to hope, that a few observations, even in this abridged manner, such as the limited room of this Journal prescribes, will be acceptable to its readers.

Suppose I should have the misfortune, not to convince every one of what I state, on account of the brevity to which I am obliged to confine myself, I am, notwithstanding comforted by the reflection, that it is always useful to consider matters, belonging to the practice of physic, in different points of view, till infallible cures shall be discovered. By these means the truth is sooner found out, than if the disease should be always treated in the same imperfect way. Many important truths, when first discovered, have met with the fate to be rejected, but, on more mature consideration, were afterwards generally acknowledged. Who does not know what adversaries Gallilleo found,

 when when he published the revolution of the Earth round the Sun; yet, is there any body who doubts it now?

Most of what I shall say of the epidemic in Spain, is, with little difference, applicable also to the plague in the Turkish states, to the yellow fever in the tropical climates, to the jungle fever in Ceylon, to the Hungarian fever, to the malignant ague epidemic in Italy, to the prison fever, to the ship fever, to the inirritative fever, to the typhus and to the low nervous fever, because all these denominations signify only different gradations or modifications of the same disorder, which manifests itself by symptoms of putridity, as they are commonly called. Amongst others, by a great prostration of strength; by derangement of the faculties of the mind; by blood, less apt to coagulate, when out of the vessels; by several appearances of the skin, viz. petechiæ, a change of its natural colour into a yellow or lead hue; a dry heat in the beginning, which feels unpleasant to the observer, and in the more advanced state, clammy and sometimes cold sweats.

The peculiarity, that all the above named fevers attack at the same time the generality of the inhabitants of a province, of a town, of a ship, or of a prison, convinces us, that its cause also must be a general one, and of a nature to influence all the inhabitants in a similar manner, because they all suffer alike. It cannot therefore consist in the diet, neither in the exercise, nor in the manner of dressing, nor in keeping regular or irregular hours, nor in the state of mind, nor in many other things, which are so differently observed by a whole public; though we remark at the time of an epidemic, a general and uniform aberration from the healthy state in all the inhabitants, or at least with so small differences, as additional causes, like uncleanliness, subterraneous habitations, &c. can easily explain.

Consequently we conclude, that the cause of so general and violent a distemper, exists in a circumstance which,

1. Is equal to its effect.
2. Which influences equally all classes of the inhabitants; and,
3. Of the greatest consequence for regulating the degrees of animal life; that is to say, to cause either good health, illness, or death.

Let us then examine, whether we might find in the atmosphere a cause, equal to, and in sufficient connection with, the effect, so as to be convinced that this cause is more more satisfactory to a philosophic mind than any other that can be assigned.

It is certain, and an axiom in natural philosophy, that nothing in the whole of nature has a more general, sudden, uniform, and greater influence upon all classes of mankind, than the atmosphere; and that nothing is more essential to the continuance of health and life, than its qualities. The great influence of the atmosphere upon the human constitution is not only confined to breathing, but is manifest also by its effects upon the whole surface of the body in the following ways:

1. By pressure.
2. By imbibing the particles of the insensible perspiration.
3. By being partly absorbed by a set of vessels, distributed for this purpose over the whole skin.

Every practitioner, whatever his knowledge in Natural Philosophy may be, is aware, that certain alterations in the atmosphere produce certain alterations in our constitution, which we signify by the general denomination, DISEASE; and specify them according to the symptoms which most strongly strike us, by particular names, as plague, ague, rheumatism, dysentery, cough, &c. Or, is there any person who does not know, that rheumatism, cough, and pneumony arise after continued north-east winds? Colds, and diarrhœas, after cold and damp weather? the first has even got the name from its cause. Typhus and dysentery after a hot summer? Ophthalmy after too bright an atmosphere? Chilblain after frost? Diminished sense of hearing in too rarefied an atmosphere?

I also call the attention of the reader to another fact, which elucidates in a great degree, what I wish to prove; I mean, that because some changes in the atmosphere happen pretty regularly in certain seasons, also some diseases make their appearance regularly in the same seasons. To this class can be reckoned vernal and autumnal agues; the plague in Egypt, when the atmosphere has imbibed numberless exhalations from the overflowing of the Nile. Cough and colds in the autumn. Dysentery and typhus in the latter end of the summer. Pneumony and sore throat in the winter.

Another strong proof of the remarkably great influence of the state of the atmosphere upon our constitution, we find in such diseases as are peculiar to particular countries, and which originate mostly from particular qualities of the atmosphere in those countries. This is in a number of instances instances so accurate, that we could undertake to write a pathological geography: In which, Egypt would be the country of Ophthalmy; the Turkish dominions, of the Plague; Low Hungary, of the Hungarian Fever; the Antilles Islands, of the Yaws; Barbadoes, of Elephantiasis; the West Indies, of the Yellow Fever; America, of Syphilis; Ceylon, of the Jungle Fever; England, of Asthma and Gout; Holland, of Ague; Swisserland, of Bronchocele and Cretins; Poland, of Plica Polonica; Lombardy, of Pellagra; the North Sea, of Scurvy. But these examples, I presume, are sufficient to prove, that a particular state of the atmosphere produces particular diseases, corresponding to this state.

This being proved, let us now consider:

1. What is the particular state of our atmosphere proper to preserve health and life? and,

2. What alterations happen, capable of causing indispositions, or an aberration from good health?

To the first question, I answer, that an atmosphere, fit for breathing health and life, ought to consist of seventy parts of nitrogen, twenty-seven of oxygen, and commonly three of carbonic acid gas. This contains nearly also the answer to the second question; namely, 1. That each variation from this particular state is productive of some one or other disease, even of death, if the oxygen gas is altogether nearly wanting. 2. That each addition of strange aeriform fluids renders the atmosphere more or less fit for respiration and productive of diseases, according to their quality or their quantity, by which they diminish the proportion of oxygen. Their quality, like sulphuric vapour, is sometimes so baneful as to end life immediately.

After having endeavoured to convince by what has been said, that different states of the atmosphere have the power either,

1. To continue animal life; or,

2. To end it; or,

3. To produce diseases:

We turn our attention to the symptoms of the epidemical fever in Spain, and of the epidemics in general, which are nearly related to that one; and we shall soon be persuaded that they are produced by want of oxygen in the animal body.

Oxygen being the cause of excitement, which is wanting in this malady to a degree not sufficient to produce either appetite, or to perform voluntary motions, or some natural functions: and also, the blood not having the florid colour which proceeds from oxygen, and when out of of the body, becoming putrid some hours sooner than the blood of a healthy man; the skin losing its natural colour, and taking a yellowish red, or other hue; the strength being totally prostrated, the faculties of the mind disturbed, the external senses weakened and inactive, the blood so thin that it extravasates from the mouths of the vessels, causing both petechiæ and nose-bleeding; the carbon exhaled from the lungs, not being sufficiently united with oxygen, precipitates both upon the tongue and teeth;* the abundant carbon in the excrements, which manifests itself by their black colour, and the eagerness of the patient for acid things are, sufficient reasons to conclude:

"That the *next cause* of the epidemic in Spain is want of oxygen in the constitution."

But the great source of oxygen in the animal body being the atmosphere, from which it is taken both by the lungs and skin, we may with equal reason conclude, "that the *remote cause* of the epidemic in Spain, consists in an undue proportion of the component parts of the atmosphere."

To elucidate this way of arguing further, it may be proper to convince the reader by some symptoms of the distemper, that an alteration in the atmosphere alters also its effect *upon the surface of the animal body.* For, we observe on the skin a burning heat, clammy cold sweats, petechiæ, discoloration, and a constricted state of it; symptoms which lead us to judge fairly, that the usual absorbing powers and pressure of the atmosphere are diminished; and being in want of oxygen, it absorbs this substance more eagerly from the surface of the body than usually. From this particularly proceeds the great and sudden mortality of the yellow fever and plague, because the patient does not only breathe enough of a substance so essential to the continuance of animal life, but loses rapidly what he has, through the exhalent vessels of the skin; neither can the absorbent vessels recover it from an atmosphere where there is nothing to be got. From this loss originates also the yellow colour of the skin, because oxygen bleaches solid parts in acidifying them.

We must not pass by in silence an additional demonstration of an empirical remedy, administered to avoid the pernicious influence of the atmosphere upon the skin; I mean,

* In a state of good health, a part of the oxygen which is inspired, is again expired, saturated with carbon, forming carbonic acid gas.

I mean oiling the whole body; a remedy which has acquired a great name amongst the remedies recommended against the plague, and has lately also been made use of against the epidemic fever in Spain. The Hungarian shepherds were long acquainted with it, as a preventive remedy against the Hungarian fever; they oil their shirts, and are sure to escape the distemper by this means, though they are, during many months, night and day, exposed to the open air. What other effect can this remedy produce than to prevent the oxygen from eagerly passing through the skin, to supply the atmosphere with what it is in want of? A similar circle we observe throughout the whole of nature. A cold body gives a part of its heat to a warmer one; a dry atmosphere absorbs the humidity from the wet soil, and lets the water fall again to the dry soil, &c.

The reader having been convinced by what has been said, that want of oxygen in the constitution is the next proximate cause, and an undue proportion of the component parts of the atmosphere the remote cause of the epidemy in Spain; perhaps it may be asked, what has produced this undue proportion of the component parts of the atmosphere particularly this year? And in what does this alteration consist? This I shall now endeavour to elucidate, that I may remove all difficulty.

We are acquainted with no operation in nature which causes so violent, manifold, and sudden effects, as subterraneous fire, not only by its expansive powers, but by its developing enormous masses of aeriform fluids, which finding no room in the internal parts of the earth, must make its way more or less violently into the atmosphere, according to the degree of opposition. Perceiving at the very first view some relation between this cause and the effect we have now spoken of, we shall examine it more minutely, to see, whether it will prove sufficiently satisfactory.

But, before I inquire farther into this circumstance as a cause of the corrupted atmosphere, I think it incumbent upon me to prove, that gases, especially such as are pernicious to animal life, do really pierce through the surface of our globe, and mix with the atmosphere, which is best done by facts, as the shortest and best demonstrative mode. One of the most striking instances of this sort we see in the Grotta del Cane near Naples. The man who opens the door, makes, for the entertainment of the traveller, the cruel experiment; which proves strongly, that a gas, unfit for for respiration, pierces through its bottom, and impregnates the atmosphere. He puts the nose of a dog near the bottom of the Grotta, where the pernicious gas is in a small degree diluted with atmospheric air, and the poor victim falls within a minute into convulsions, and dies if he is not brought immediately to the atmospheric air again, where he revives, to be exposed another day to the same pneumatic experiment, which after four or five repetitions, ends its life irrecoverably. The gas piercing thro' the bottom of this Grotta is, as been proved by experiments, the carbonic acid gas, developed by the fire of Vesuvius.

After the eruption of this mountain in 1794, several inhabitants at Portici, and in the neighbouring villages, lost their lives in going rashly into their cellars, by suffocation, occasioned by carbonic acid gas, which had pierced through the bottom.

At the foot of the volcano it happens frequently, that hunters lose dogs, as they pass, in their course, a stream of the beforementioned fatal gas. Asses likewise, when grazing, fall now and then a victim to it.

The same gas, originating from the same cause, is found under the antient theatre at Pompeij, and in some places of France; also at Pyrmont, not far from the well of the mineral water, is a spot, where birds, in passing it, drop down dead, on account of this same gas piercing through the ground.

Classic authors mention the same fact above the surface of the Lago d'Averno, which, however, is not the case in our days. Virgil says,

"Ubi venere ad fauces graveolentis Averni."

And the same author, in another place,

"Quam super haud ullæ poterant impune volantes

"Tendere iter pennis, talis sese halitus atris

"Faucibus effundens supera ad convexa ferebat

"Unde locum Graij dixere nomine Aornon."

This fact appears remarkable, in so far as it proves that gasses piercing through the bottom of the sea may come to its surface, and show different effects according to their quantity, and the violence with which they are forced up. Does it not throw some light upon the origin of hurricanes?

These facts are convincing that aeriform fluids, especially carbonic acid gas, arise through the surface of the earth, and lowering the state of the atmosphere must be productive of diseases. We are now naturally led to the question, question, by what means the carbonic acid gas is developed, and mixed with the atmosphere? We see two principal causes to effect this.

1. Either mere heat, which by heating calcareous matters, expels the carbonic gas, contained always in great abundance in these substances. (A process we imitate in making quick lime.) Or,

2. Sulphur (one of the most common nourishments of subterraneous fires) after being burnt, is converted into sulphuric acid, which coming into contact with calcareous substances, expels likewise all the carbon with which it is saturated.

If this new produced gas is generated in enormous quantities, which cannot find an easy outlet, but must force its way with violence through the surface of the earth, it makes fissures and cracks, of which earthquakes are the consequence. May not this also be the origin of hurricanes?

It remains, lastly, agreeably to my purpose, to examine, whether there have been appearances, which prove a greater activity of subterraneous fire this year, than in those years in which no epidemy of that kind has prevailed? By which fire, according to what we have said before, a greater mass of carbonic acid gas, and of other aeriform fluids, unfit for respiration, have been forced into our atmosphere. That this has been the case, I shall prove by some select and convincing instances.

1. Vessuvius has been active to a considerable degree, and has thrown out uncommonly great quantities of lava, after a perfect repose of ten years.

2. Earthquakes have been very frequent in Spain, in Tuscany, in the ecclesiastical state, in Guernsey, at Kingston, at Sumatra, at Mantura, and in many other places.

3. Hurricanes in the West Indies have been more violent than usual.

4. Near the fortress Phanagoria in Russia, there was a considerable volcanic eruption in July last; a new mountain rose,* which, when it came to the height of twelve fathoms, burst with a noise like thunder, threw out a gas of a disagreeable smell, ignited stones, &c. and a part of the lake near to it appeared dry.

5. Superficial

* A phenomenon we are already acquainted with; the *Monte Nuovo*, near Naples, is another instance of it. Also, whole islands risen from the sea.

5. Superficial earth fires have been observed in several places.

After these quoted facts, no person will doubt any longer of the unusual great activity of subterraneous fires during this year, which cannot exist without developing enormous volumes of carbonic gas, because nothing is more common than calcareous fossils, which always contain carbon. I think that this, and every other circumstance, is now so minutely explained, that every body will consider himself entitled to conclude: "That the remote cause of the fever in Spain, Marseilles, and Leghorn, is the fatal superabundant addition of carbonic acid gas to the atmosphere, originating from the direct or indirect effect of fire upon calcareous substances in the bowels of the earth." The truth of which is still more strengthened by a negative way of arguing; I mean, by not being able to find out another cause, which answers equally well this extraordinary and sudden effect of such extent, violence, and duration.

Or, if any person doubts, without good argument, what I have stated, I request him to try, whether he can discover another cause, that will give more satisfaction.

Before I proceed to the cure, I call the reader's attention to the phenomenon of the ignis fatuus in marshy countries, as an instance also, that other gases are found as noxious additions to the contents of the atmosphere. The ignus fatuus is hydrogen gas, which burns when it comes in contact with the atmosphere, and is most common in countries frequently visited by agues.

### CURE.

After having demonstrated the cause of the epidemy, it is now incumbent upon us to propose a cure, which opposes both the proximate and remote cause; and being founded upon the removal of it, there is full reason to expect that this mode of treatment would always answer. And it certainly would, if only the method could be found out of making the stomach bear the *quantity* of the remedy adequate to the degree of the distemper.

To elucidate this proposition, I quote a fact, that every practitioner succeeds in curing intermittent fevers of little resistance. But those, who are afraid of giving several ounces of the bark in the course of twenty-four hours, do not cure the obstinate ones, though they administer the proper remedy, only not in a sufficient dose. Would the sufficient sufficient quantity of bark for curing obstinate quartan-autumnal fevers, injure the stomach, as a *too large dose* of vitriolic acid does, we should leave them uncured, as we have till now left the plague and yellow fever.

In order to oppose the cause of this epidemy (which should be the object of every rational cure), we must endeavour to supply the constitution with a sufficient quantity of oxygen, and prevent the escape of what remains in the body. For obtaining the first point, nothing is equal to

*Mineral Acids,*

Because no article in the materia medica contains in the same bulk more oxygen, therefore no other remedy can answer the purpose equally well. But as in the higher degrees of the distemper, viz. plague, yellow fever, and in the worst cases of the reigning epidemy in Spain, the stomach cannot bear the quantity necessary to effectuate the cure, it is of great importance to administer, together with the acids, other remedies, which answer the same end.

1. Give from thirty to one hundred drops of the vitriolic acid, diluted with barley water, and repeat it several times in the day, according to the degree of the malady. The best way to take it, is to suck it through a tube, to avoid setting the teeth on edge. I recommend especially the hyper-oxygenated muriatic acid, because it separates easiest from its super-abundance of oxygen.

2. Cover the whole body with ice or snow, and where that cannot be had, throw cold water over the patient, or cover his whole surface with camphor, made into an ointment with eggs, or any other substance which dissolves it well.

3. Make the sick breathe oxygen gas, mixed with atmospheric air in a proportion adequate to the degree of the disease.

4. Administer clysters of dissolved crystallised acid of lemons, or of vinegar, to patients of the lower class. Give only from three to four ounces at a time, that it may remain in the rectum, and become in part absorbed.

5. Genuine wine should be the common beverage. Rhenish is the best; next to it, port. Strong good porter may also be taken with advantage.

6. Let the patient drink frequently, but not too much at once, as the beverage would go off too quickly by the urine.

7. Expose the sick to a constant draught of air, and let the the room be cool, for cold air being more condensed, the same bulk must contain more oxygen than in a rarified state. Bring the patient into the open air, especially upon an elevated situation. Carry him in open carriages, waggons, or chairs. Let him ride on horseback, if he has strength for it. He breathes by these means oftener, changes the atmosphere, and meets in consequence with more oxygen.

8. Keep the sick room clear of every thing which may lower its atmosphere. Remove the night stool, the foul linen, &c. and let neither animals nor men be in the room, the nurse excepted.

9. Wash the sick room, bedstead, &c. with lime water, and expose it in large flat vases to the atmosphere to absorb the carbon of it, with which this fluid has a great affinity, as the surface shows after a short time.

10. The sick ought to occupy the largest room in the house.

11. Let the sick rooms in private houses, or wards in the hospital, be fumigated according to Dr. Carm. Smyth's method, in order to provide oxygen.

12. Fumigations with juniper berries, several kinds of woods, resins, &c. are not only of no use, but even prejudicial, by impregnating the atmosphere with vapours unfit for respiration. All these substances cause but a good smell, and deceive people if they believe that, from this circumstance, the atmosphere is also improved for respiration.

13. I need not say, what every body ought to know, that bleeding and purging are dangerous.

14. What the sick eats or drinks must be as cold as it can be procured, even icid, if it can be obtained.

These appear to me the principal rules for effecting the cure of the epidemical fever in Spain. Should they not prove successful in the worst cases, a number of lives, however, which would inevitably have been lost by improper treatment, like purging, blistering, or bleeding, will be preserved by this method. And as soon as ways and means can be found out to increase the doses of acids, the cure of that distemper will be as certain as that of agues.

There remains now another task, of equal interest to the public, namely, to shew how to preserve the health of those not yet attacked by the disease. The methods to be adopted must, of course, differ a little (though not in essential points) according to the epidemic, the climate, the local local circumstances, &c. and ought, in regard to these, to be applied with discernment.

### *Proposals to prevent the Progress of an Epidemic.*

1. In the first days, when the epidemic makes its appearance, object to no person who is in good health quitting the place. Only, if they go at a late period, after their oxygen is in a great measure spent, and they do not employ efficacious remedies to recover it, they must die at the place whither they retire; perhaps even sooner, as they exhaust themselves by precipitate and anxious journies. If they steal away, and go by night, on foot, in bad weather, by round about ways, through rivulets, marshes, and bushes, in order to elude discovery, they must fall ill, as a consequence of this imprudence and over fatigue. Leaving the town in the *very beginning* of the epidemic is of equal benefit, both to the healthy and to the sick, because fewer inhabitants consume less oxygen. This measure is especially of use in fortified towns, where the circulation of the air is interrupted by walls and ramparts, and rendered insalubrious by the stagnating water in the ditches.

2. If a cordon be already formed, permit at least the inhabitants of the town to abandon their dwelling houses, and to inhabit the highest mountains in their reach. Because the carbonic gas, on account of its greater heaviness, remains more or less in the under regions of the atmosphere, and the circulation is livelier upon the heights.

3. Remove dogs, monkies, and all superfluous animals from the place of the epidemic.

4. Let the people no where be crowded. In churches and theatres they must not remain long, otherwise the air becomes unfit for respiration, and produces the malady.

5. Pay particular attention to the cleanliness of the lower class of people, and to the Jews, who incline, in general, to dirtiness; which is not only hurtful to themselves, but also to their neighbours.

6. Should the epidemic happen in a maritime situation, send all classes of inhabitants, of all ages, and both sexes, to the sea to bathe; or to the river, if it happens in inland places. This can easiest be executed in a garrison.

7. Order the soldiers out of the barracks into tents, placed upon heights.

8. Allow no salt provisions, and let the people live more on vegetable than animal diet.

9. Procure large quantities of lemons and oranges, and give them to the poor, gratis.

10. The market for vegetables must be well stocked; especially with such of them as contain much sugar, viz. carrots, beet-root, parsnips, &c. and they should be sold at a cheap rate.

11. Roman Catholics must get indulgence from the church not to observe their fast days, especially in hot countries; where I often remarked the fish not to be sweet in the market.

12. Soldiers ordered on night duty, ought to take a glass of brandy in the evening.

13. Restrain the night duty as much as possible.

14. Advise and order the inhabitants to change their linen oftener than they commonly do.

15. Foul linen is to be washed immediately, because its exhalations are pestiferous.

16. Engage the minds of the people by public amusements. Let the military bands play more than usual; allow and invite rope-dancers, and all kinds of shows, to enter the town, in order to make the inhabitants forget their melancholy situation.

17. Suffer the principal persons of the place by no means to leave their station. Let them be actively employed in procuring relief of all kind; but they must appear gay and unconcerned, in order to fill the mind of the inferior classes with confidence; because the common people are guided in their opinion by the behaviour of the superior ranks.

18. Let genuine porter be sold under its price. The public should make up the loss to the publicans, or, which would be still better, sell it themselves, to have it genuine.

19. Order the churches, barracks, theatres, dwelling-houses, hospitals and prisons to be fumigated, according to Dr. Carm. Smyth's prescription.

20. Recommend gymnastic plays of all kinds.

21. The beds of the deceased must not be burnt in the town, because it lowers the state of the atmosphere, and makes a melancholy impression upon the mind, especially in small places, where every body is aware of the cause of the fire.

22. Each man, before he touches a corpse, is to take a glass of brandy, or of wine.

23. Publish and distribute gratis, rules for the healthy. Warn them, amongst others, to keep regular hours, to take sufficient exercise, to bathe daily, to air the rooms well, to keep every thing particularly clean, to wash and sprinkle the house and furniture with lime water, to be moderate in eating, to consume much fruit, to drink wine freely, to take strong lemonade instead of tea, to prevent animals, like dogs, monkeys, &c. from entering the dwelling houses, to retire in tents upon the mountains, to keep gay company, &c. &c. &c.

24. Provide enlightened clergymen and priests, who should discourage all despondency, else the patients will often not listen to medical advice, being impressed with the idea that they must die, and they must only prepare for this event. The sick are converted to pure fatalism by the ill management of the monks, of which I saw a number of instances in Italy. One of my patients at Rome was forbid by a capuchin to take the emetic, prescribed on the day he had taken the communion.

25. Each nurse, who attends patients, must be allowed a bottle of wine per day. The expence can be of no consideration, if human life is to be saved by it.

26. If lime can be procured, let the deceased, when buried, be covered with it.

27. By no means, and under no pretext, must any body be buried in churches or vaults.

28. The tombs ought to be made at a considerable depth, and superintended by public authority.

29. Do not over fatigue servants, soldiers, journeymen, &c.

An exact and judicious application of these means, with perhaps such additions as the local circumstances may render necessary, will be found effectual in stopping the progress of the distemper, and preserve the healthy from it.

I come now to the last task, to propose such measures as may be likely

*To prevent the Return of similar Epidemics.*

The rules to obtain this end differ considerably, according to the nature and cause of the epidemic, to accustomed diet, to local circumstances, &c. so that, properly speaking, they could be given only for a particular epidemic, and for a particular place and public, which, however, would lead me too far for my present purpose, and for this limited ed space. Besides, it would be out of my power to give infallible rules for towns whose peculiar situations, and other particularities, which cause or influence the epidemic, I am not acquainted with. I shall explain this by some examples. In Egypt, the canals of the Nile must be cleaned, and brought into order, to prevent the return of the plague. In Hungary, the marshes must be drained and cultivated, to prevent the return of the Hungarian fever. This is the same in the Pontine marshes, which are not drained with sufficient skill, because the smaller ditches, joining in right angles with the principal one, obstruct the flow of the water in the principal canal. In long voyages, to prevent scurvy, the sailors and passengers must be very punctual in bodily cleanliness, eat much sour crout, drink lemon acid, and take bodily exercise.

Before we give particular rules for the prevention of an epidemic, we must beg leave to say a few words about the quarantine, whose laws appear not yet well regulated. In some cases, it is certainly an unnecessary interruption to commerce and travellers, and becomes hurtful when precautions at home are through it neglected. By what philosopher's calculation is it, that the infectious matter preserves itself forty days in ships which arrive from a harbour where any epidemic is reigning? Or, who has ascertained that the poison of the plague preserves its infectious nature as long as that of the yellow or epidemical fever in Spain? Has the length of the voyage no influence upon the powers of the infectious matter? Why is a ship from Jamaica kept equally long in quarantine as another from Spain? How is it to be ascertained, when the power of infection is over? Some physicians, who practised in the West-Indies, do not allow the infectious nature of the yellow fever? Suppose all ships from Constantinople, from the whole Levant, the Mediterranean, and from the West-Indies, were to perform the strictest quarantine before Egypt, the country still will not be free from the plague, till the ancient canals are restored. In former times, when the canals were in proper order, the plague was not known in that country; on the contrary, it was reckoned so healthy, that the Roman physicians, according to Celsus's account, used to send patients thither for the recovery of their health. If that country became a permanent province of a European power, the eradication of the plague may probably be expected, as the first benefit for the inhabitants.

In some countries, the inhabitants have found out their own remedy to escape an epidemic. They abandon some

 villages villages in the Campagna Romana, and the houses of the Pontine marshes, during some of the summer months, because they know by a long experience, that remaining where they are, they fall victims to malignant agues. In some instances, they go to a neighbouring village, at a small distance, situated only higher, and find perfect safety. This last mentioned epidemic originates, in all probability, from an addition of hydrogen gas to the atmosphere, which takes its origin from marshes, stagnating waters, and putrefaction of vegetables.

We come now to the preventive rules, and propose in general, that in Spain, which an epidemic ravages so frequently, the land should be manured, by public order, with lime, because it absorbs eagerly the carbon from the atmosphere, and parts with it again to the vegetables, growing in the same soil. The lime having parted with this substance, soon absorbs it again from the atmosphere, and parts with it again, by which circle it becomes a lasting remedy, which promotes at the same time vegetation, because plants want nothing more for their growth than carbon. This is also the reason, that in some places in Italy, where no man can preserve his health during the summer, the vegetation is extremely luxuriant, because plants find that principle in abundance, which promotes their growth, and injures animal life.

If all the African towns, which the plague often depopulates, have such narrow streets as I saw at Tetuan, (through many you cannot go on horseback, on account of their narrowness) they ought to be built wider, and strait, and the windows, or rather holes, for letting in air and light, must be larger. Besides this improvement, procure European physicians, who are philosophers, to be able to give proper advice in the beginning of the plague, in order to stop its spreading, and to devise a method to prevent the return of the calamity.

Are the Spanish towns kept equally dirty as what I saw Lisbon? the first object ought to be, the introduction of a better police. The metropolis of Portugal, on account of its dirty streets, wherein all filth, chamber-pots and night-stools not excepted, are thrown, would have every year a similar epidemic, if it were not prevented by its high airy situation, and by the flowing of the Tagus, both which cause a constant change of atmosphere, so that when hardly impregnated with the exhalation of the filth, it is caried to other regions, especially by the lasting north winds uring the summer.

Build

Build in all towns near the sea or rivers, where epidemics make their appearance, subterraneous drains, to carry off immediately all kind of dirt and filth, before it corrupts by its exhalations the state of the atmosphere.

Let the new buildings be superintended by a public architect, to have them built airy. The doors and windows must not be on the same side, as I have seen at Gibraltar, as, in this case, the air of the room cannot be sufficiently renewed. Attention to this circumstance is the more necessary, as people in hot climates are inclined, for temporary convenience, to exclude the sun from the rooms ; not reflecting, that by preventing the air from entering, they hereby give rise to an additional cause for epidemics ; a proceeding which ought therefore to be prevented by public authority.

The inhabitants of Gibraltar have frequently committed the error of excluding the access of fresh air to their dwelling houses, being limited much for room to build upon.

Many people must not sleep in the same room, and the chamber-pots must be cleaned immediately in the morning. In Lisbon you find the whole family sleep upon the floor, in the same room in which they live and dine. I entered sometimes in the morning such rooms, before the windows were opened, and was in danger of being suffocated.

Air prisons and ships carefully, wash them with lime water, and fumigate them according to Dr. C. Smyth's method.

On the prevention of the return of the distemper, much more may be said than I have advanced in these pages ; but to lay down rules with any benefit, they should only relate to a particular place, which would lead me too far.

Though much more may be observed on this subject, so very interesting to human happiness, I feel myself obliged here to conclude this Essay, as the place in which it appears does not allow of prolixity.

I should be happy and fully rewarded for my present labours in the cause of those who are suffering by an epidemic, if the adoption of these proposals should be the means of affording them relief.

